# Modafinil Improves Catalepsy in a Rat 6-Hydroxydopamine Model of Parkinson’s Disease; Possible Involvement of Dopaminergic Neurotransmission

**DOI:** 10.15171/apb.2017.043

**Published:** 2017-09-25

**Authors:** Reza Vajdi-Hokmabad, Mojtaba Ziaee, Saeed Sadigh-Eteghad, Siamak Sandoghchian Shotorbani, Javad Mahmoudi

**Affiliations:** ^1^Department of veterinary, Miyaneh branch, Islamic Azad University, Miyaneh, Iran.; ^2^Medicinal Plant Research Center, Institute of Medicinal Plants, ACECR, Karaj, Iran.; ^3^Neurosciences Research Center (NSRC), Tabriz University of Medical Sciences, Tabriz, Iran.; ^4^Department of Immunology, Tabriz Branch, Islamic Azad University, Tabriz, Iran.

**Keywords:** 6-hydroxydopamine, Dopaminergic neurotransmission, Modafinil, Parkinson’s disease, Rat

## Abstract

***Purpose:*** Modafinil is a vigilance-enhancing drug licensed for narcolepsy. The use of modafinil leads to various neuromodulatory effects with very low abuse potential. A body of evidence suggested that modafinil may have anti-parkinsonian effects. This study was designed to evaluate whether modafinil could improve motor dysfunction in the 6-hydroxydopamine (6-OHDA)-induced rat model of Parkinson’s disease.

***Methods:*** Male Wistar rats (180-220 g, n= 98) were used in this study. Parkinsonism was induced by injection of 6-hydroxydopamine (10 μg/2μl in 0.2 % ascorbic acid-saline) into the right striatum. Parkinsonian rats received intraperitoneal (ip) injections of modafinil (50, 75, and 100 mg/kg) and catalepsy-like immobility was assessed by the bar test (BT). Furthermore, involvement of dopamine D_1_ and D_2_ receptors in modafinil’s anti-parkinsonian effects was studied. For this purpose, parkinsonian animals were pretreated with SCH23390 and raclopride (the dopamine D_1_ and D_2_ receptor anatgonists, respectively) or SCH23390 + raclopride, and then assessed by the BT.

***Results:*** Modafinil (100 mg/kg) showed anti-cataleptic effects in the BT. Notably, the effect of modafinil in the BT was reversed in parkinsonian rats pretreated with raclopride (1.25 mg/kg) and/or SCH23390 + raclopride (0.75 and 1.25 mg/kg, respectively), but not in those pretreated with SCH23390 (0.75 mg/kg).

***Conclusion:*** Acute administration of modafinil improves 6-OHDA-induced motor impairment possibly through activation of dopamine D_2_ receptors.

## Introduction

Parkinson’s disease (PD) is the second most common neurodegenerative condition characterizing with motor symptoms including akinesia, bradykinesia, tremor at rest, rigidity^1^ and leads to extensive biochemical and molecular alterations in cerebral structures that are involved in motor function.^2,3^ Dopamine (DA) regulates normal motor activity through D_1_ and D_2_ receptors that are found postsynaptically on the dopaminergic (DAergic) neurons^4^ in the striatum.^5^ Studies showed that degeneration of the nigrostriatal pathway alters the brain’s D_1_ and D_2_ receptor densities.^6,7^ Such changes play a compensatory role and may consider as a promising therapeutic target in PD.^8^

L-DOPA (3,4-dihydroxyphenylalanine) restores DA level to normal value, but responses to this regimen decline over the time and the patients experience some motor abnormalities.^9^ Hence, development of effective therapies to manage PD complications is of great interest.

Modafinil is a vigilance-enhancing compound^10^ first approved by the Food and Drug Administration for treatment of sleep disorders such as narcolepsy, shift-work sleep disorder and obstructive-sleep apnea syndrome.^11,12^ Because of its complex and wide spectrum pharmacologic profiles, there are efforts for its application in conditions such as nicotine and cocaine addiction,^11^ schizophrenia, memory impairments, depression^13^ and PD.^10,14^ Results of positron-emission tomography (PET)^15^ and micro-dialysis^16^ studies have shown that modafinil has the ability to increase cerebral DA levels. Given the above, modafinil appears to provide anti-PD effects via modulation of DAergic neurotransmission. Therefore, the present study was set out to evaluate modafinil’s anti-parkinsonian effects in a rat model of PD and the involvement of D_1_ and D_2_ DAergic receptors in this effect.

## Materials and Methods

### Animals

Ninety eight male Wistar rats weighing 180-220 g were used for the experiment. Animals were kept under controlled conditions (12/12 h light /dark cycle: lights on at 07: 00 hours, ambient temperature 21±1°C, humidity 55±5%) with unrestricted access to food and water.

### Drugs and treatments

All chemicals were purchased from Sigma-Aldrich Chemical Co. (USA). For systemic administration, modafinil was suspended in saline with 0.4% sodium carboxymethyl cellulose. SCH23390 (the DA D_1_ receptor antagonist) and raclopride (the DA D_2_ receptor antagonist) were dissolved in distilled water. 6-OHDA was dissolved in a 0.9% normal saline solution containing 0.2% (w/v) ascorbic acid. Drugs were freshly prepared and injected intraperitoneally (ip) in a volume of 1 ml/kg body weight, except for 6-OHDA which was injected into the right striatum. Desipramine (25 mg/kg, ip) was injected 30 min before intra-striatal injection of 6-OHDA, in order to prevent the destruction of noradrenergic neurons.^17^

Two sets of experiments were performed in this study. The first experiment was conducted to assess modafinil’s ability to reduce the immobility time in parkinsonian animals. In this phase, rats with 6-OHDA lesion received different doses of modafinil (50, 75, and 100 mg/kg) or its vehicle and then, after 30 minutes, were subjected to the bar test (BT).

The second set was carried out to evaluate the possible involvement of the DAergic system on the anti-immobility effect of modafinil in the BT. In this phase, individual groups of parkinsonian animals were pretreated with SCH23390 (0.75 mg/kg, ip), raclopride (1.25 mg/kg, ip) and/or both of these (or their vehicles) at the same doses in combination, and after 30 minutes, received modafinil (100 mg/kg) or the its vehicle. The doses of antagonists used in this study was approximately the same as that reported by Hauber et al.^18^

### Intra-striatal injection of 6-OHDA

For stereotaxic surgery, animals were anesthetized with a combination of ketamine and xylazine (80 and 5 mg/kg, ip; respectively) and placed in a stoelting stereotaxic apparatus (stoelting, USA) in the flat skull position. The small central incision was made to make the skull appear. A 23 gauge sterile cannula was inserted into the injection site as a guide cannula for subsequent insertion of the injection tube into the striatum. The coordinates for this position, with reference to the atlas of Paxinos &Watson,^19^ were: anteroposterior from bregma (AP)= 0.4 mm, mediolateral from the midline (ML)= 2.8 mm and dorsoventral from the skull (DV)= -5 mm. Subsequently, 6-OHDA (10 μg/ rat in 2 μl saline containing 0.2% ascorbic acid) was infused by an infusion pump at the flow rate of 0.2 μl/min into the right striatum. Lesioned rats were subjected to the designed protocols after a 3-week recovery period. All of these procedures were performed for sham-operated animals, but they only received intra-striatal of 2 μl vehicle of 6-OHDA (0.9% saline containing 0.2% (w/v) ascorbic acid).

### Assessment of catalepsy-like immobility 

Catalepsy-like immobility was assessed by using BT. As described previously, both forelegs of a rat were gently placed over a 9-cm-high horizontal bar (diameter, 0.7 cm) and the retention time in this imposed posture was considered to define catalepsy time. The end point of catalepsy was designated to occur when both front paws were removed from the bar or the animal moved its head in an exploratory fashion. The cut-off time of the test was 180 seconds.^17,20^

### Verification of infusion site

To verify the infusion site, all rats were sacrificed by a high dose of ether at the end of behavioral assessments. Afterwards, the brains were removed and stored in 10% formaldehyde solution for one week prior to embedding and sectioning. Serial coronal sections (6 μm) were taken with a microtome (Leitz, Germany) and stained with haematoxylin-eosin; the scar tract made by the infusion tube was controlled with a light microscope. Whenever the emplacement of the infusion tube in striatum was incorrect, the representative data were excluded.

### Statistical analysis

Statistical analysis of each data set was done by SPSS 21 software. The data were expressed as the mean ± SEM and were analyzed by two-and/or one-way ANOVA and post hoc Tukey’s test. *P* values < 0.05 were considered to be statistically significant.

## Results

### Effect of 6-OHDA on the BT

**Figure 1:**
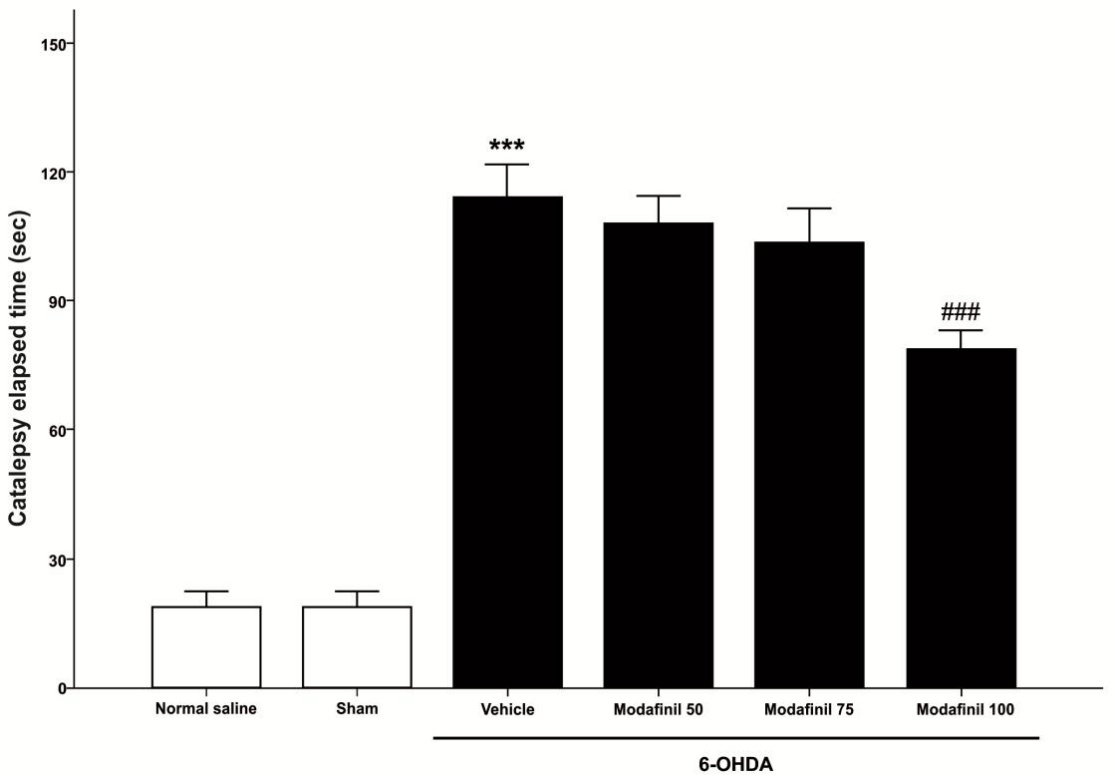
Effect of intraperitoneal (ip) injection of vehicle and/or different doses of modafinil (50, 75 and 100 mg/kg) on the 6-OHDA (10 μg/2 μl/rat)-induced catalepsy. Each bar represents the mean ± SEM. (n = 8)^***^p<0.001 and ^###^*p*<0.001 as compared with the normal saline and vehicle received groups, respectively.

One-way ANOVA revealed a significant effect of intra-striatal injection of 6-OHDA [*F*(3,28)=375.27 *p*<0.001] on the catalepsy time in comparison with control and sham-operated groups. Post hoc analysis showed that 6-OHDA (10 μg/ rat) increased catalepsy time in the BT, which indicates that this neurotoxin is able to produce marked catalepsy. Also, there was no significant difference between the sham-operated group and control rats (Figure 1).

### Effect of modafinil on the BT

One-way ANOVA showed that modafinil could attenuate catalepsy time in 6-OHDA-lesioned rats [F(3,28) = 375.27 *p*<0.001]. Post hoc analysis indicated that modafinil only at the dose of 100 mg/kg is able to decrease the immobility time in the BT when compared with vehicle-treated 6-OHDA-lesioned rats. At lower doses (50 and 75 mg/kg), modafinil has not significant effect on the catalepsy time (Figure 1).

### Effect of raclopride and SCH23390 pretreatment on the anti-cataleptic effect of modafinil

Modafinil (100 mg/kg, ip) reduced catalepsy (*p*<0.001) and the involvement of the DAergic neurotransmission on this effect was studied in separate groups of 6-OHDA-lesioned rats.

A two-way ANOVA revealed significant differences of modafinil treatment [F(1,28) = 55.3 *p*<0.001] but not SCH23390 pretreatment [F(1,28) = 0.8 *p*>0.05]. Also, there was significant differences of modafinil treatment interaction with SCH23390 pretreatment [F(1,28) = 18 *p*<0.001].

**Figure 2:**
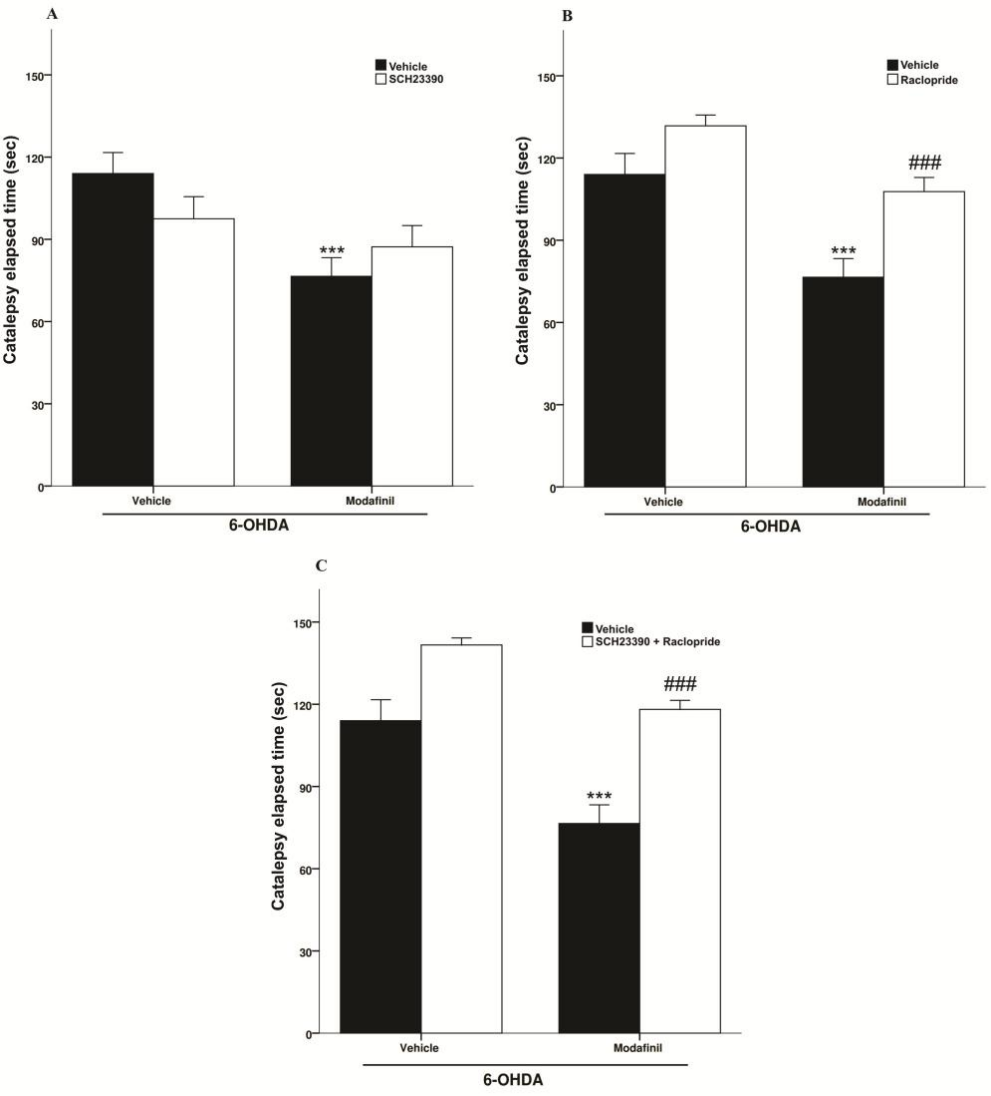
Effect of pretreatment with SCH23390 (0.75 mg/kg) (A), raclopride (1.25 mg/kg) (B) and/or SCH23390 + raclopride (0.75 + 1.25 mg/kg, respectively) (C) on the modafinil anti-cataleptic effect . Each bar represents the mean ± SEM. (n = 8)^***^p<0.001 and^###^*p*<0.01 as compared with the vehicle and the modafinil (100 mg/kg, ip) injected rats, respectively.

The results presented in in Figure 2A, show that pretreatment of lesioned rats with SCH23390 (0.75 mg/kg, ip) did not alter the anti-cataleptic effect of modafinil in the BT.

A two-way ANOVA showed significant differences of modafinil treatment [F (1, 28) = 143.7 *p*< 0.001)], raclopride pretreatment [F (1, 28) = 91.2 *p*<0.001] and modafinil treatment interaction with raclopride pretreatment [F (1, 28) = 6.92 *p*<0.05].

The results presented in Figure 2B show that pretreatment of lesioned rats with raclopride (1.25 mg/kg, ip) reversed the anti-cataleptic effect of modafinil in the BT.

A two-ANOVA revealed significant differences of modafinil treatment [(1, 28) = 169.8 *p*<0.001)], SCH23390 + raclopride pretreatment [F (1, 28) = 218.9 *p*<0.001] and modafinil treatment interaction with SCH23390 + raclopride pretreatment [F (1, 28) = 8.94 *p*< 0.01]. The results depicted in Figure 2C show that pretreatment of lesioned rats with SCH23390 + raclopride (0.75 and 1.25 mg/kg, respectively, ip) blocked anti-cataleptic effect of modafinil in the BT.

## Discussion

Our data showed that modafinil displays an anti-parkinsonian effect on the 6-OHDA lesioned rats, and this effect in part is mediated through DAergic neurotransmission.

Catalepsy or tonic immobility is a complex motor inhibition^21^ in which rodents are unable to correct externally imposed abnormal posture^21,22^ and revert to a normal position for initiation of exploratory behavior.^23^ This behavior not only is able to mimic the state of akinesia and rigidity occurring in PD^24^ but also is used to evaluate nigrostriatal function and its regulation by different neurotransmitter systems.^25^ 6-OHDA is frequently used for chemical denervation of DAergic neurons^26^ and those rats with DAergic lesion show marked catalepsy;^27^ as a result, this neurotoxin provides simple and a reliable model for studying the anti-parkinsonian potential of drugs.^20^

In this study, a single dose of modafinil (100 mg/kg) resulted in decreased catalepsy time and normalized motor behavior in parkinsonian rats 30 min after ip injection. Pharmacokinetic findings suggest that modafinil reaches a peak concentration in brain 30 to 60 min by single systemic administration^28^ and produces a rapid and significant elevation in brain DA content in dose dependent fashion.^29^

Nucleus accumbens which receives DAergic inputs from the ventral tegmental area and medial substantia nigra regulates normal motor function.^30,31^ Modafinil increases DA efflux in this region^32^ through inhibition of DA transports^16^ as well as reduction of accumbal GABAergic tone. The inhibitory effect of modafinil on the GABAergic system also enhances the activity of the striatopallidal pathway.^33^ This pathway governs normal motor function and is involved in the appearance of PD motor signs.^34^

In another portion of this study, parkinsonian rats pretreated with concomitant administration of D_1_ and D_2_ receptor antagonists (raclopride and SCH23390, respectively). This intervention increased immobility time in the BT and prevented the anti-parkinsonian effects of modafinil. Furthermore, blockade of D_2_ receptors using raclopride reversed the anti-parkinsonian effect of modafinil in 6-OHDA lesioned rats.

Studies on the striatal D_2_ receptor suggested that denervation of DAergic neurons by 6-OHDA might increase D_2_ receptor densities from 2-8 weeks post-lesion in impacted animals.^35^ Indeed, significant up-regulation of post synaptic D_2_ receptor binding sites is accompanied by elevation of D_2_ mRNA levels in 6-OHDA lesioned rats.^36,37^ Moreover, postmortem studies in drug-naive PD patients have also confirmed such increase in striatal D_2_ receptor binding sites.^38^

Contrary to D_2_ receptors, there is contradictory evidence about D_1_ receptor alterations in parkinsonian rats.^35-39^ While there are no reports showing that alteration in D_1_ density happens in PD,^38^ Zhao et al. showed that a decline in mRNA levels for D_1_ receptors in DA-lesioned striatum occurs in parkinsonian rats.^7^ This reflects that denervation of DAergic structures is not able to increase D_1_ receptors densities.^35^

Decline in striatal DA levels causes an imbalance in striatal functions and disrupts normal motor activity. Hence, pronounced up-regulation of D_2_ receptors may potentiate responsiveness to decreased levels of striatal DA and normalize motor activity,^8^ especially in *de novo* and young parkinsonian patients.^40^ Moreover, when compared with D_2_ receptors, D_1_ receptors have less ability to increase locomotor activity.^41^ Collectively, these data can explain why D_2_ receptor activation may in part mediate anti-parkinsinian effects of modafinil.

Complications such as development of abnormal motor fluctuation and inadequate responses to standard anti-parkinsonian drugs remain major problems in parkinsonian patients.^20,42^ In addition, non-motor comorbidities such as depression^43^ and sleep disorders^44,45^ are experienced by the majority of patients and impact their daily living activities.

Hence, application of regimens to overcome these problems is of great importance; the ability of modafinil to reduce PD symptoms in experimental models, as well as its potential for anti-depressant-like properties in preclinical research and treatment of sleep disorders in PD, suggests that modafinil may have potential to improve the effectiveness of current anti-PD medications.

## Conclusion

In conclusion, this study showed that modafinil improves catalepsy behavior in a rat model of PD. Considering the role of DAergic neurotransmission in regulation of normal motor behavior and alterations of D_2_ receptor densities in PD, it may be suggested that modafinil exert the anti-PD effect through modulation of DAergic system. Moreover, the complexity of modafinil’s mechanism of action suggests that more experiments must be designed to reveal its neuropharmacological effects.

## Acknowledgments

We would like to express our special gratitude to the Miyaneh branch of Islamic Azad University, Iran, for financial support.

## Ethical Issues

The experiment was performed in accordance with the Guide and Use of Laboratory Animals (National Institutes of Health) and confirmed by the Ethical Committee for Animal Experimentation of the Miyaneh branch of Islamic Azad University.

## Conflict of Interest

The authors declare no conflict of interests.
